# Outcomes of lung transplantation in adults with bronchiectasis

**DOI:** 10.1186/s12890-018-0634-4

**Published:** 2018-05-22

**Authors:** Jodie Birch, Syba S. Sunny, Katy L. M. Hester, Gareth Parry, F. Kate Gould, John H. Dark, Stephen C. Clark, Gerard Meachery, James Lordan, Andrew J. Fisher, Paul A. Corris, Anthony De Soyza

**Affiliations:** 1 Institute of Cellular Medicine, Newcastle University, M2060 Leech Building, The Medical School, Framlington Place, Newcastle upon Tyne, NE2 4HH UK; 2 Sir William Leech Centre for lung research, The Freeman Hospital, High Heaton, Newcastle upon Tyne Hospitals NHS Foundation Trust, Newcastle upon Tyne, NE7 7DN UK; 3 Department of Medical Microbiology, The Freeman Hospital, High Heaton, Newcastle upon Tyne Hospitals NHS Foundation Trust, Newcastle upon Tyne, NE7 7DN UK; 4 Institute of Transplantation, The Freeman Hospital, High Heaton, Newcastle upon Tyne Hospitals NHS Foundation Trust, Newcastle upon Tyne, NE7 7DN UK

**Keywords:** Transplantation, Bronchiectasis, Pseudomonas

## Abstract

**Background:**

Lung transplantation is a well-established treatment for end-stage non-cystic fibrosis bronchiectasis (BR), though information regarding outcomes of transplantation remains limited. Our results of lung transplantation for Br are reported here.

**Methods:**

A retrospective review of case notes and transplantation databases was conducted for patients that had underwent lung transplantation for bronchiectasis at the Freeman Hospital between 1990 and 2013.

**Results:**

Fourty two BR patients underwent lung transplantation, the majority (39) having bilateral sequential lung transplantation. Mean age at transplantation was 47.1 years. Pre-transplantation osteoporosis was a significant non-pulmonary morbidity (48%). Polymicrobial infection was common, with *Pseudomonas aeruginosa* infection frequently but not universally observed (67%). Forced expiratory volume in 1 second (% predicted) improved from a pre-transplantation mean of 0.71 L (22% predicted) to 2.56 L (79 % predicted) at 1-year post-transplantation. Our survival results were 74% at 1 year, 64% at 3 years, 61% at 5 years and 48% at 10 years. Sepsis was a common cause of early post-transplantation deaths.

**Conclusions:**

Lung transplantation for end-stage BR is a useful therapeutic option, with good survival and lung function outcomes. Survival values were similar to other bilateral lung transplants at our centre. Pre-transplantation *Pseudomonas* infection is common.

## Background

Bronchiectasis is an abnormal dilation of the bronchi and bronchioles that results in chronic cough, sputum production and recurrent infections. Bronchiectasis can lead to progressive loss of lung function, resulting in chronic morbidity and premature mortality [1]. Bronchiectasis not due to cystic fibrosis (often referred to as non-cystic fibrosis bronchiectasis; thereafter BR) has diverse causes, though post-infectious and idiopathic bronchiectasis are the most common [2, 3].

BR has been identified as a cause of increasing morbidity and mortality in the U.S. and Europe [4–6]. As Bronchiectasis is increasingly encountered (or recognised) there is a greater need to understand the benefits and risks of lung transplantation for this indication. Lung transplantation is an intensive therapeutic intervention that can be performed for the treatment of end-stage BR [7, 8]. However, the recent guidelines from the British Thoracic Society (BTS) specifically note scarce data on the results of lung transplantation for bronchiectasis [2]. This knowledge gap results in uncertainty for clinicians in managing patients with more severe bronchiectasis.

A number of studies have assessed the association between pathogenic microorganisms and prognosis in adult BR. Persistent *Pseudomonas aeruginosa* infection is seen in approximately 30-40% of BR patients and it is linked with a poorer quality of life and increased mortality [9, 10]. Furthermore, it predicts a more severe disease phenotype with increased hospitalisation rates and is associated with poorer lung function and accelerated functional decline in BR patients [9–12]. In some settings, *Pseudomonas* infection post-transplantation has been linked with increased rates of allograft dysfunction/obliterative bronchiolitis [13]. In contrast, information regarding the prognostic effects of pre-transplantation *Pseudomonas* status on both early and long-term outcomes of lung transplantation for BR remains limited.

In view of the above, we aimed to assess the survival outcomes of patients transplanted for BR at our centre. Additionally, we aimed to investigate a range of pre-transplant factors including pre-transplantation microbiology and their relationship to post-transplantation outcomes.

## Methods

Our primary outcome of interest was post-transplant survival in those transplanted for BR. Other aims were to describe the demographic profiles of patients transplanted and the post-transplant outcomes in patients with BR as compared to other lung transplant indications

### Case finding and definitions

A retrospective analysis of the pulmonary transplantation databases and case notes was performed for all BR patients who underwent pulmonary transplantation at our institution from 1990 to 2013. All adult recipients with bronchiectasis as a primary diagnosis were assessed and their case notes and microbiological results reviewed. In general, the exclusion of cystic fibrosis was through genetic testing by UK Health service genetic laboratories and/or sweat tests in line with more recent guidelines. Immunological work up included assessment of serum immunoglobulins although additional tests were performed upon consultation with immunologists if clinical suspicion of immunodeficiency was made.[2] As a control group we included all lung transplants for any other indication across the same time cohort. Data where available were extracted to define the Bronchiectasis severity index scores [4], the FACED scores [14] and the eFACED scores [15].

Peri-transplantation management

Induction therapy changed over the time cohort but has included intravenous methylprednisolone and in earlier patients included anti-thymocyte globulin [16]. A 3-day induction protocol with intravenous methylprednisone (2 mg/kg) was used in the majority of patients. Post-transplantation immunosuppression comprised of cyclosporine, prednisolone and azathioprine for all patients [16]. Prophylactic antibiotics were given to the recipient in accordance with the most recent sensitivities derived from sputum cultures as per our CF protocol [16]. Aztreonam (2 g) 8 hourly for 2-7 days was used if the isolate was multiply resistant. Multiple antibiotic synergy testing has been incorporated since 2001 into our microbiological work up using previously described methods [17, 18].

### Operative interventions

Bilateral single sequential lung transplantations (BSLTx) were performed via clamshell incisions as per our CF lung transplant protocol [16]. The donor bronchial stump was kept as short as possible to avoid ischaemic injury. Cardiopulmonary bypass was used in all cases with aprotinin used as standard. Heart-lung transplantation was performed *via* sternotomy with tracheal anastomosis and bicaval anastomosis.

### Surveillance associated complications

Surveillance transbronchial biopsies and bronchoalveolar lavage (BAL) were routinely performed at 1 week, 1 month, 3 months, 6 months and one year post-transplant and at times of deterioration [16]. Acute vascular rejection grade A2 or greater were recorded. Major complications of transbronchial biopsy were recorded as present if there was requirement for chest drain insertion, biopsy associated bleeding with requirement for invasive ventilation or death following a procedure [16].

### Obliterative bronchiolitis

Pulmonary function tests were performed according to accepted guidelines. The data were collected prior to the use of chronic allograft dysfunction in clinical practice [19] so Bronchiolitis obliterans syndrome terminology was used. We used “Freedom from BOS” as previously [20] to define patients who failed to demonstrate a fall in FEV_1_ to the threshold used for BOS 1 or higher. The best consecutive FEV_1_ attained as directed by the guidelines was used to set thresholds for BOS 1 (FEV_1_ 66-80% of best recorded post-transplantation FEV_1_) BOS 2 (FEV_1_ 51-65%) and BOS 3 (FEV_1_ <50%). BOS 0-p (potential for BOS development) was also recorded.

### Survival analysis and causes of death recording

Survival data are routinely collected as part of the national transplant surveillance programme. StatView software V.4.5 was used to conduct actuarial survival analysis within our cohort. Causes of post-transplantation mortality were recorded from patient notes where available. Sepsis related deaths were recorded where a pathogen was identified clinically as causal to the recipient’s death or where a clinical diagnosis of infection was made and alternate diagnoses were excluded.

### Microbiology

Peri-transplantation microbiology from sputum and/or BAL of the recipient lung on the day of transplantation was recorded from patient notes and the microbiology database. In most cases sputum was collected with infrequent need for BAL at transplant. Pre-transplantation sputum microbiology results e.g. from referring centres or at our transplant assessment visits were also recorded from patient notes. The presence/absence of bacterial infection was based on qualitative microbial culture. No quantitative cultures were undertaken. Pulsed field gel electrophoresis assessment of microbiological clonality was not routinely conducted. Post-transplantation BAL data from routine surveillance BAL undertaken at 1 year was cross-checked between the computerised pathology reporting system and from paper records.

### Systemic disease

Pre-transplantation cardiac dysfunction, body mass index (BMI) and osteoporosis rates from Dual-energy X-ray absorptiometry (DEXA) scans were captured. Post-transplantation renal function was determined by serial serum creatinine levels that were recorded pre-transplantation and at 1 year, 5 years and 10 years post-transplantation.

## Results

The total number of lung transplantation procedures performed for all indications at data capture (1990-2013) was 752, with 42 lung transplantations performed for BR (6% of the total lung transplant population). There were 39 patients that underwent BSLTx from cadaveric donors, one patient had single lung transplantation (SLTx) and two patients had heart-lung transplantation. Lung transplantation commenced at this institution in 1987 with the first transplantation for BR performed in 1990. The assessment protocol has evolved in this time period and hence full datasets are not available for all parameters.

There were 25 patients transplanted for BR between 1990 and 2000 from a total of 260 lung transplants performed (9.6%). Significantly fewer were transplanted between 2001 and 2011; 17 from a total of 429 (4.0%; Chi-square test, p<0.001). Thus, lung transplantation for BR was less frequent in the second 10-year time cohort compared to the first 10 years of transplantation. All recipients were adults (age >17 years), with a mean age at transplantation of 47.1 years (range; 22.6-62 years). There were 13 female patients (31%) and 29 male patients (69%) transplanted. For the control cohort (all sequential single lung transplants performed for any other indication) the mean age was 42 years with 42% female and 58% male (the majority of these other indications were cystic fibrosis and COPD without bronchiectasis).

### Bronchiectasis aetiology

Bronchiectasis aetiology was categorised in 29 of 42 patients, with post-infectious (9 patients), idiopathic (6 patients) and COPD-associated (5 patients) accounting for the majority of cases (31%, 21% and 17% of cases, respectively).

Bronchiectasis associated with Kartagener’s syndrome was noted in 14% of cases (4 patients) and Young’s syndrome in 10% of cases (3 patients). Neonatal ventilatory trauma and X-linked agammaglobulinaemia led to secondary bronchiectasis in single cases. In the remaining cases the presumptive aetiologies were idiopathic or post infectious but insufficient details were available to conclusively exclude other aetiologies.

### Bronchiectasis severity scores

Full data sets were not available in all transplant recipients. We were able to calculate the BSI, FACED and eFACED scores in 34 patients. According to the BSI, 33 had severe bronchiectasis (score 9 or greater) and one had moderate severity (BSI score 7). In contrast whilst 18 were deemed to have severe bronchiectasis 16 were deemed to have moderate bronchiectasis according to FACED. Using eFACED scores, 28 were deemed to have moderate bronchiectasis and 8 were deemed to have severe bronchiectasis.

### Pre-transplantation morbidity

#### Pulmonary disease

The mean pre-transplantation FEV_1_ at lung transplant assessment was 0.71l ± 0.27 (22 % predicted) (*n* = 37). From 36 patients where full data was available, 32 were in respiratory failure (89 %) and on long-term oxygen therapy. Of these, one patient was on long-term non-invasive ventilation (NIV).

Pre-transplantation arterial blood gas values taken at assessment were available for 35 patients. The mean PO2 was 8.3 ± 2.8kPa and mean pCO2 was 6.9 ± 1.2kPa (30/35 patients on oxygen therapy). Six minute walk test mean distance walked was 280.8 metres (range; 60-640 metres), with lowest arterial oxygen saturation recorded at a mean of 75.4% (range; 49-91%) (n = 36).

Data regarding other co-morbidities were available for 31 of 42 patients. Of these, we noted 12 patients (39%) with a co-existent diagnosis of asthma pre-transplantation.There were 10 patients (32%) with COPD. Five were felt to have COPD as an aetiology and five patients had another diagnosis felt to be the primary cause of the bronchiectasis but also had COPD listed as a comorbidity. A history of previous pneumothorax was reported by 4 patients (13%). We noted 3 patients (10%) with echocardiographic evidence of pulmonary hypertension and 2 patients (6%) with clinical features of allergic bronchopulmonary aspergillosis.

#### Non-Pulmonary disease

Osteoporosis was a significant non-pulmonary pre-transplantation morbidity affecting 14 patients (48%) (*n* = 29). 3 patients had documented pre-transplantation diabetes (10%, *n*=31). Pre-transplantation echocardiogram results were available for 33 patients. Of these patients, 18 (55%) had an abnormal result, with some patients having multiple abnormalities. Right ventricular dilation was noted in 13 cases, right ventricular atrophy in 4 cases, left ventricular dilatation noted in 2 cases and pulmonary hypertension noted in 1 case. Pre-transplantation ischaemic heart disease affected 2 patients (6%) and Wolff-Parkinson-White syndrome affected one patient. At assessment the pre-transplantation mean BMI was 25.4 kg/m2 (± 4.8) (range; 16-31.9 kg/m2) (*n* = 25).

#### Survival and causes of death

**Fig. 1:**
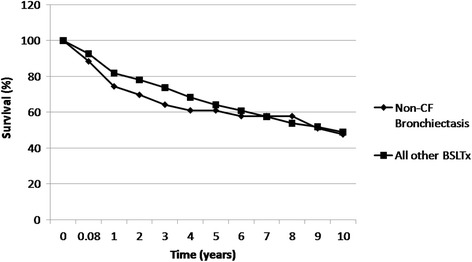
Actuarial survival of patients with non-CF bronchiectasis (BR) at the Freeman Hospital Lung Transplant Programme (*n*=42) compared with all other bilateral sequential single lung transplants (BSLTx) performed at our centre (*n*=409). No significant difference in survival was found between the cohorts (log-rank testing; Mantel-Cox, *p* = 0.23).

The survival figures for the whole cohort were 74% survival at 1 year, 64% at 3 years, 61% at 5 years and 48% at 10 years (Fig. 1).,The calculated 50% survival was at 9.3 years. We compared our Kaplan-Meier survival rates for the BR cohort with that of BSLTx for all other transplantation indications at our centre (Fig. 1). There was no significant difference in survival between the BR and non-BR transplantation cohorts (log rank testing; Mantel-Cox, *p* = 0.23).

At data capture, 14 of 42 BR lung transplant recipients were alive (33%). Data allowing determination of cause of death was available in 13 cases (Table 1). Death caused by sepsis was noted in 5 cases (staphylococcal infection was identified as a cause of death in two cases and cytomegalovirus in one case). In the remaining two cases, a sepsis syndrome was identified although no specific pathogen was isolated. Therefore 38% of all recorded BR transplantation recipient deaths were due to sepsis where data were available. Of the sepsis-related deaths, 3 occurred early post-transplantation. None of these occurred in patients with known immunodeficiency related bronchiectasis. Multi-organ failure occurring within the first month following transplantation was the cause of death in 2 cases. Later causes of death included respiratory failure or obliterative bronchiolitis, which was recorded in 4 cases. Other identified causes of death include malignancy (*n* = 1, post transplantation lymphoproliferative disease) and cerebrovascular accident (*n* = 1).

**Table 1:** Causes of death in non-CF bronchiectasis recipients transplanted at the Freeman Hospital

*Cause of death*	*No of patients (%)*
Infection	5 (38)
*Staphylococcus*	2 (15)
Cytomegalovirus	1 (8)
Unknown	2 (15)
Respiratory failure	2 (15)
Obliterative bronchiolitis	2 (15)
Multi-organ failure	2 (15)
Malignancy	1 (8)
Cerebrovascular accident	1 (8)
Total No of deaths	13 (100)

Transplant Programme. Causes of death in recipients were recorded from case notes. Data are expressed as percentage of deaths observed in this cohort where data detailing cause of death were available. Data was available for 13 patients, however 28 patients from the cohort were deceased. Percentages have been rounded up or down to the first decimal place

#### Pulmonary function post-transplantation

Mean pre-transplantation FEV_1_ was 0.71l ± 0.27 (22% predicted) (*n* = 37), which improved to 2.56l ± 1.02 (79 % predicted; *n*=31) at 1 year post-transplantation. The mean FEV_1_ at 5 years post-transplantation was 2.3l ± 0.95 (74 % predicted) (*n* = 18) and 2.36l ± 0.72 (78% predicted) (*n* = 9) at 10 years post-transplantation (*p* <0.001 at each time point compared with pre-transplantation values; paired t-test).

The prevalence of severe airflow limitation as BOS 3 was 18% at 1 year and 25% at 5 years. Where data were available at 10 years post-transplantation, no patients were at stage BOS 3 (*n*=9).

#### Renal disease

Mean serum creatinine for patients at pre-transplantation assessment was 83.2 mg/dl (± 17.4) (range; 53-118 mg/dl) (*n* = 39). By 1-year post-transplantation, creatinine levels worsened for all patients, rising to a mean of 166.8 mg/dl (± 60.2) (range 73-281 mg/dl) (*n* = 29) (*p* <0.001; paired t-test). Of those patients still alive at data capture, no patients had however required haemodialysis or had undergone renal transplantation following lung transplantation (*n* = 14).

#### Surveillance biopsies

Acute vascular rejection (grade A2 or greater, as defined by the International Society for Heart Lung Transplantation (ISHLT)) [21] was noted in 2 patients from available transbronchial biopsy results at 3 months and 6 months post-transplantation (*n* = 14). Of all patients alive at data capture, none had experienced significant morbidity (e.g. invasive mechanical ventilation or blood transfusion) or mortality following standard transbronchial biopsy procedures at the unit.

#### Microbiology

**Fig. 2:**
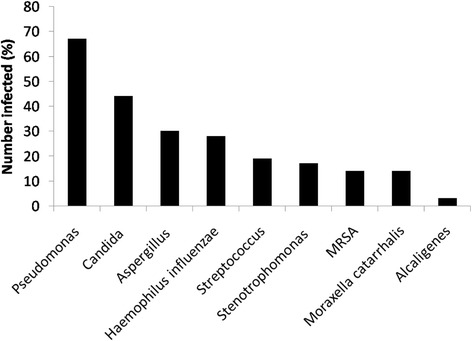
Microbial infections prior to transplantation. Percentage of the cohort (where data were available) infected with each pathogen is noted. The majority of patients had more than one pathogen isolated from the same individual’s sputa in the year before transplantation. MRSA, methicillin resistant S aureus.

Peri-transplantation microbiological cultures, including those at or before transplantation assessment, immediately pre-operatively and at 1 year post-transplantation were carried out. Polymicrobial infections in individual recipients were common (Fig. 2). We noted 67% of patients (where data were available, *n* = 36) had documented history of infection with *P. aeruginosa* before transplantation assessment. At transplant assessment, 62% of patients were infected with *P. aeruginosa* (*n* = 34) and at time of transplantation 45% of patients were infected with *P. aeruginosa* (*n* = 37). None of the patients in this cohort had infection with pan-resistant *P. aeruginosa,* however 45% of patients were recorded as having previous infection with multi-resistant *P. aeruginosa* (*n* = 20).

Other organisms commonly isolated pre-transplantation include a mixture of probable commensals and pathogens: *Candida* was noted in 44% of patients, *Aspergillus* spp. in 30%, *Haemophilus influenzae* infection in 28%, *Streptococcus pneumoniae* in 19%, *Stenotrophomonas* spp. infection in 17%, *methicillin resistant Staphylococcus aureus* (MRSA) infection in 14%, *Moraxella catarrhalis* in 14% and *Alcaligenes* spp. infection in 3% (*n* = 36).

Prior to transplantation 24 patients (69%) of 35 patients with available data, were taking nebulised antibiotics. Only 4 patients were receiving azithromycin pre-transplantation, perhaps reflecting the more recent widespread use of macrolides in inflammatory lung disease.

#### Post-transplantation lavage

Microbiology results for BAL specimens collected at 1 year post-transplantation were retrieved for 29 patients of the 31 recipients alive at 1 year. Most did not grow respiratory pathogens in their BAL (18 patients, 62%). The most commonly isolated pathogen was *P. aeruginosa* in 6 patients (21%), all of whom had persistent *P. aeruginosa* infection prior to transplantation. Other organisms isolated included *Candida* species (3 patients; 10%), *Staphylococcus aureus* (2 patients; 7%), *Aspergillus fumigatus* (1 patient; 3%) and *Paecilomyces lilacinus* (1 patient).

## Discussion

Despite exciting new therapeutic pipelines in BR, a rising mortality rate and increasing hospitalisation rates for BR suggests there are significant unmet medical needs [6, 9]. Lung transplantation is one option for managing severe end stage BR. Lung transplantation for BR accounts for 6% of all lung transplantations performed at our centre, a distribution similar to that of the International Society for Heart Lung Transplantation (ISHLT) registry [21, 22]. We noted excellent post-transplant outcomes with greater than 50% survival at 5 years. Our outcomes were comparable to lung transplant outcomes for other indications at our centre. Our approach has predominantly been with BSLTx, which has been argued as the procedure of choice for this group of patients [21, 22]. Notably BR has been calculated to have a better cost effectiveness outcome following lung transplantation as compared to COPD, the commonest indication for lung transplantation [23]. The combined UK transplant experience also suggested that BR has one of the best post-transplant outcomes [24].

In view of this, the low rates of lung transplantation for BR need to be considered. They may be due to a number of factors including concerns about the risk benefit ratio of lung transplantation in BR. The prevalence of BR peaks in older patients who may be perceived beyond the optimal window for lung transplantation. Furthermore the lack of a validated prognostic scoring systems for BR in contrast to those for COPD may prevent timely referral for lung transplantation [25]. The role of either of the recently published indices, Bronchiectasis severity index (BSI) [4] or FACED score [14] in guiding transplant referrals remains to be defined. We noted that the majority of patients were classified as severe bronchiectasis using BSI but not with FACED. Further studies are needed to define the role of these scores in helping prompt referral for transplant assessment. These latter scores underrepresent those with severe disease until the age is beyond 70 years due to the weighting of the scores.

As highlighted in recent BR guidelines very few studies have examined lung transplantation in detail. The survival in our series was similar to the survival figures previously reported by our institution for CF patients [16].

The most recent case series of 34 patients from Germany reported good outcomes for bronchiectasis with one-year Kaplan-Meier survival for patients with bronchiectasis being 85% and 5-year survival being 73%. These outcomes were comparable to the overall lung transplant cohort. Notably however the mean age group was much younger at 40 years. In those with pre-transplant Pseudomonas infection poorer outcomes and higher rates of BOS were reported from the Hannover group [26]. The UK wide experience spanning 5 centres with 123 BR patients listed for transplantation was noted in a study of all lung transplant indications published in 2009 [24]. Unfortunately, little in depth data beyond survival in BR were available but the study demonstrated only 54 BR patients listed survived on the waiting list to transplantation (48%). Of those transplanted the median waiting time on the list was nearly 1 year with a median post-transplantation survival of 3000 days. Notably the BR post-transplantation survival was the best of 5 major indications for lung transplantations. Despite this apparently good outcome it seems unlikely that the BR cohort were less sick than the other indications studied; along with interstitial lung disease, BR had the highest “on-list” pre-transplantation mortality rates (59/123 died on the waiting list). This correlates well with our observed high rates of respiratory failure and secondary pulmonary hypertension in our cohort.

The ISHLT registry data show that the major causes of mortality in the first year following lung transplantation for any indication are graft failure and infection. We noted a large range of pathogens with potential to complicate the early postoperative period. Our immediate pre-transplantation rate of *Pseudomonas* infection was 45%, which is broadly similar to a prior Spanish series of 17 patients where 64% of patients had *Pseudomonas* infection pre-transplantation [27]. These contrast with our experience in CF, where the majority of patients had *Pseudomonas* pre-transplantation [16].

BR transplant recipients could be predicted to suffer high rates of infection or, in the event of over-cautious immunosuppression, high rates of acute rejection. Firstly we observed an early septic death rate of 7%, which appears similar to that observed elsewhere for other non-septic lung transplantation [16]. Whilst there were high rates of multi-resistance in those with *Pseudomonas* infection, none were pan-resistant. Furthermore, the septic deaths were unrelated to *Pseudomonas* infection *per se*. The prior literature denotes that *Pseudomonas* infection is seen in those with more severe bronchiectasis, which has led other authors to conclude that it is a marker of more severe lung disease. It is plausible that a non-significant trend towards more deaths in the *Pseudomonas* group herein reflects more severe disease. Alternatively, as suggested by Rademacher and colleagues in the Hannover series, Pseudomonas may be driving poorer outcomes [26].

The previously noted UK wide BR transplant data set of 54 patients is likely to include many of the 37 BR patients transplanted at Papworth Hospital, reported in a case series in 2005 [28]. In this cohort, 32 were defined as “bronchiectasis alone” and the remaining 5 had an antibody deficiency that required immunoglobulin replacement therapy. In this latter case series the observed actuarial survival was similar in the 2 groups (81% at 12 months in the bronchiectasis group and 80% in the antibody deficiency group). The post-operative complication rates were acceptable with infection episodes per 100 patient-days for bronchiectasis alone being 0.90 vs. 0.53 and rejection episodes per 100 patient-days being 0.59 vs. 0.24. Whilst we did not quantify rejection rates in this manner, our rates of symptomatic or surveillance rejection were not detected.

Whilst this is the largest single centre study of lung transplantation in bronchiectasis to date limitations of our study should be acknowledged. These include missing data points: whilst there were 28 deaths we could only report data on 13 of these cases reflecting that many of the deaths occurred late transplant and occurred at the referring centre and not transplant centre. The newer definition of chronic lung allograft dysfunction (CLAD) [19] was not used in our study awaiting ISHLT guidelines on the implementation of CLAD. The study is limited by the sample size that are inherent in the single centre retrospective design. Furthermore there have been changes in both our peri transplant protocols reflecting novel immunosuppressants and anti-viral agents used over the study period. Additionally the majority of the transplants performed are sequential single lung transplants so we cannot define the differences between heart-lung transplantation and our preferred operative type. Larger multicentre studies, with multivariate analyses, that define pre-transplant characteristics that are associated with increased risks of early deaths would be helpful. Nevertheless our study highlights important findings that have not been reported before. Important areas to be considered for future studies will be an assessment of rejection rates and frequency of and prognostic implications of clonal *Pseudomonas* strains [29].

## Conclusions

Transplantation for BR has excellent outcomes yet poor on-list survival [24]. In our experience the numbers of transplants for this indication may be declining for reasons unknown. Physicians should consider transplantation as an option in those with severe bronchiectasis.

## Abbreviations

BR: non-cystic fibrosis bronchiectasis xzBSLTx Bilateral single sequential lung transplantations BAL broncho-alveolar lavage BOS bronchiolitis obliterans syndrome BMI body mass index DEXA Dual-energy X-ray absorptiometry SLTx single lung transplantation NIV non-invasive ventilation BSI bronchiectasis severity index.
